# Transcriptomic Analysis of Seed Coats in Yellow-Seeded *Brassica napus* Reveals Novel Genes That Influence Proanthocyanidin Biosynthesis

**DOI:** 10.3389/fpls.2017.01674

**Published:** 2017-10-05

**Authors:** Meiyan Hong, Kaining Hu, Tiantian Tian, Xia Li, Li Chen, Yan Zhang, Bin Yi, Jing Wen, Chaozhi Ma, Jinxiong Shen, Tingdong Fu, Jinxing Tu

**Affiliations:** National Key Laboratory of Crop Genetic Improvement, National Sub-Center of Rapeseed Improvement in Wuhan, Huazhong Agricultural University, Wuhan, China

**Keywords:** *Brassica napus*, seed coat color, proanthocyanidin, transcriptome, transcription factor

## Abstract

Yellow seeds are a favorable trait for *Brassica* crops breeding due to better quality than their black-seeded counterparts. Here, we compared the *Brassica napus* seed coat transcriptomes between yellow- and brown-seeded near-isogenic lines (Y-NIL and B-NIL) that were developed from the resynthesized yellow-seeded line No. 2127-17. A total of 4,974 differentially expressed genes (DEG) were identified during seed development, involving 3,128 up-regulated and 1,835 down-regulated genes in yellow seed coats. Phenylpropanoid and flavonoid biosynthesis pathways were enriched in down-regulated genes, whereas the top two pathways for up-regulated genes were plant–pathogen interaction and plant hormone signal transduction. Twelve biosynthetic genes and three regulatory genes involved in the flavonoid pathway exhibited similar expression patterns in seed coats during seed development, of which the down-regulation mainly contributed to the reduction of proanthocyanidins (PAs) in yellow seed coats, indicating that these genes associated with PA biosynthesis may be regulated by an unreported common regulator, possibly corresponding to the candidate for the dominant black-seeded gene D in the NILs. Three transcription factor (TF) genes, including one bHLH gene and two MYB-related genes that are located within the previous seed coat color quantitative trait locus (QTL) region on chromosome A09, also showed similar developmental expression patterns to the key PA biosynthetic genes and they might thus potentially involved participate in flavonoid biosynthesis regulation. Our study identified novel potential TFs involved in PAs accumulation and will provide pivotal information for identifying the candidate genes for seed coat color in *B. napus*.

## Introduction

*Brassica napus* is one of the most important oilseed crops across the world which provides not only vegetable oils and biofuels for human life but also high-quality proteins for livestock feed. Breeding *B. napus* cultivars with yellow seed coats is a desirable method for improving the oil content and meal quality of rapeseed, because yellow seeds have a number of advantages that include a thinner testa, higher oil and protein contents, lower fiber and lignin contents, and reduced polyphenolics and pigments compared to their black-/brown-seeded counterparts (Rahman, 2001; Wittkop et al., 2009). As *B. napus* lacks a natural yellow-seeded resource, researchers have endeavored to resynthesize yellow-seeded cultivars/lines (such as, No. 2127-17) through the introgressions of yellow seed traits from other *Brassica* species (*Brassica rapa, Brassica juncea, Brassica carinata*, and *Brassica oleracea*) or relative genus (*Sinapis alba*), which exit naturally yellow-seeded germplasms (Chen et al., 1988; Rakow et al., 1999; Rahman, 2001; Li A. M. et al., 2012; Wen et al., 2012). The formation mechanism of seed coat color remains more elusive in *B. napus* than that in the model Brassicaceae plant *Arabidopsis thaliana* and other *Brassica* species due to multiple yellow-seeded genomic resources and the various seed coat color influenced by environment factors (Yu, 2013).

**Figure 1:**
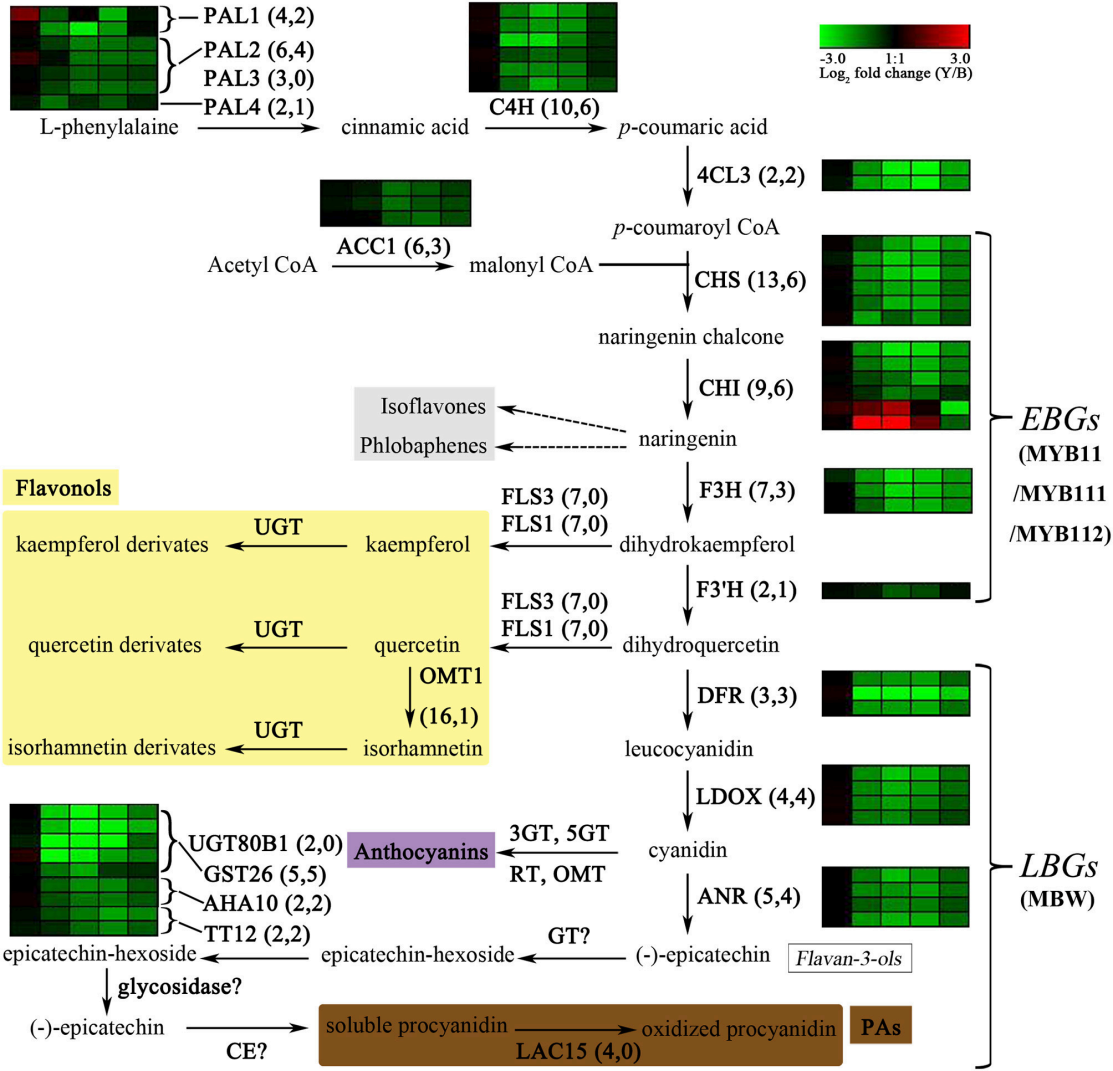
Expression changes in biosynthetic genes involved in the flavonoid biosynthesis (adapted from Auger et al., 2010 and Routaboul et al., 2012) in the yellow and brown seed coats during seed development. The copy numbers of biosynthetic genes from the whole genome and from the DEGs in the present study are listed in parentheses, respectively. Log_2_ fold changes (Y-NIL/B-NIL) of DEGs at 14, 21, 28, 35, and 42 daf are illustrated with green or red boxes ordered left to right. Green or red boxes indicate down- or up-regulation of the genes in Y-NIL, respectively. PAL, phenylalanine ammonia-lyase; C4H, cinnamic acid 4-hydroxylase; 4CL, coumaric acid: CoA ligase; ACC1, acetyl-CoA carboxylase 1; CHS, chalcone synthase; CHI, chalcone isomerase; F3H, flavonol 3-hydroxylase; F3′H, flavonol 3′-hydroxylase; FLS, flavonol synthase; OMT1, O-methyltransferase 1; DFR, dihydroflavonol reductase; LDOX, leucoanthocyanidin dioxygenase; ANR, anthocyanidin reductase; UGT80B1, UDP-glucose: sterol-glucosyltransferase; GST26, glutathione-S-transferase 26; AHA10, H^+^-ATPase 10; TT12, MATE transporter; GT, glycosyltransferase; CE, condensing enzyme; LAC15, laccase 15; *EBG*, early biosynthetic gene; *LBG*, late biosynthetic gene; MYB11/MYB111/MYB112, MYBs controlling the EBGs expression; MBW, MYB-bHLH-TTG1 complexes to regulate the expression of the LBGs. Steps that still need to be characterized are indicated in a question mark.

The seed coat color in *A. thaliana* is determined by proanthocyanidin (PA) content as shown by the analysis of a range of *transparent testa* (*tt*), *tannin-deficient seed* (*tds*), and other related mutants. PAs are synthesized as one of end products of the flavonoid biosynthesis pathway (Figure 1), which has been well described in some reviews (Lepiniec et al., 2006; Saito et al., 2013; Appelhagen et al., 2014; Xu et al., 2014). Flavonoids are derived from the general phenylpropanoid pathway, which involves three classes of enzymes, phenylalanine ammonia-lyase (PAL), cinnamic acid 4-hydroxylase (C4H), and 4-coumaric acid: CoA ligase (4CL). To date, more than 27 genes have been identified to participate in or affect flavonoid biosynthesis, mainly including biosynthetic and regulatory genes. The biosynthetic genes are divided into early and late biosynthetic genes (EBGs and LBGs, respectively). The EBGs include *chalcone synthase* (*CHS*/*TT4*), *chalcone isomerase* (*CHI*/*TT5*), *flavonol 3-hydroxylase* (*F3H*/*TT6*), and *flavonol 3*′*-hydroxylase* (*F3*′*H*/*TT7*). The LBGs include the three structural genes *dihydroflavonol-4-reductase* (*DFR*/*TT3*), *leucoanthocyanidin dioxygenase*/*anthocyanidin synthase* (*LDOX*/*ANS*/*TT18*), and *BANYULS*/*anthocyanidin reductase* (*BAN*/*ANR*), as well as three genes encoding the transporters *autoinhibited H*^+^*-ATPase isoform 10* (*AHA10*/*TT13*), *TT12* (*MATE transporter*), and *glutathione-S-transferase 26* (*GST26*/*TT19*/*GSTF12*), and a laccase gene *TT10* (*laccase 15*) (Lepiniec et al., 2006; Xu et al., 2014). Yet, the function of a gene encoding UDP-glucose: sterol-glucosyltransferase (*TT15*/*UGT80B1*) in seed coat coloring is not well-understood (Stucky et al., 2015). Furthermore, abundant modification by enzymes such as, glycosyltransferases, methyltransferases, and acyltransferases leads to the huge chemical diversity of flavonoids (Saito et al., 2013). Regulation of biosynthetic genes is orchestrated by different sets of transcription factors (TFs), covering MYBs, bHLHs, TRANSPARENT TESTA GLABRA1 (TTG1, a WD40 repeat protein), TTG2/WRKY44, TT16/AGL32 (a MADS-domain protein), and TT1 (a WIP zinc-finger protein; Xu et al., 2015). The EBGs are regulated by at least three R2R3-MYBs, namely MYB11, MYB12, and MYB111. The LBGs expression is mainly controlled by different MYB–bHLH–WDR (MBW) ternary complexes. In vegetative tissues, anthocyanin biosynthesis is regulated by different MBW complexes comprised of PRODUCTION OF ANTHOCYANIN PIGMENT 1 (PAP1/MYB75), PAP2/MYB90, GLABRA3 (GL3/bHLH001), ENHANCER OF GLABRA3 (EGL3/bHLH002), TT8 (bHLH042), and TTG1. In seeds, PA biosynthesis is mainly controlled by the TT2-TT8-TTG1 complex, while three additional MBW complexes (MYB5–TT8–TTG1, TT2–EGL3–TTG1, and TT2–GL3–TTG1) were shown to function in a tissue-specific manner (Xu et al., 2014). In addition, the MYB113, MYB114, MYBL2, MYB4, CAPRICE (CPC), TRIPTYCHON (TRY), SQUAMOSA PROMOTER BINDING PROTEIN-LIKE 9 (SPL9), SEEDSTICK (STK/AGL11), and LATERAL ORGAN BOUNDARY DOMAIN (LBD) families and some microRNAs also negatively regulate flavonoid biosynthesis (Dubos et al., 2010; Gou et al., 2011; Mizzotti et al., 2014; Sharma et al., 2016).

Many studies of flavonoids composition and *TT* homologs have shown that the flavonoid pathway is also involved in the seed coat color formation in *Brassica* species (Akhov et al., 2009; Auger et al., 2009, 2010; Li et al, 2010; Liu et al., 2016). Flavonols and PAs are the major pigments accounting for the seed coloring of *A. thaliana* and *Brassica* (Lepiniec et al., 2006; Auger et al., 2010; Routaboul et al., 2012). Flavonols are present in both the seed coat and the embryo. Colorless PAs [polymers of 3-cis-(-)-epicatechin (flavan-3-ols)] that accumulate exclusively in the inner integument, are oxidized and polymerized into brown pigments by TT10 during seed maturation, leading to darkening of the seed coat (Xie et al., 2003; Zhang et al., 2013). The flavonoid pathway in *Brassica* species is much more complex than that in *A. thaliana* because of more flavonoid-related genes and multi-loci interactions due to genomic polyploidization (Yu, 2013; Liu et al., 2016; Qu et al., 2016a). Homologs of some flavonoid-related genes have been cloned and characterized in *Brassica*, such as, *PAL1, DFR, BAN, TTG1*, and *TT1* (Ni et al., 2008; Akhov et al., 2009; Auger et al., 2009; Zhang J. F. et al., 2009; Yu, 2013; Lian et al., 2016).

The seed coat color in the resynthesized yellow-seeded *B. napus* line No. 2127-17 is mainly controlled by four loci, *D, Y, B*, and *C*. The genes *D, B*, and *C* are the dominant black-seeded genes; *Y* is a partially dominant yellow-seeded gene (dominance relationships: D > Y > B = C). One major quantitative trait locus (QTL) (*Bnsc-9a*: locus *D*) in linkage group N9 was identified in an F2 population developed from the cross of No. 2127-17 with the black-seeded line 94570 (Zhang Y. et al., 2009; Zhang et al., 2011). To identify the candidate for gene *d* in No. 2127-17, we have developed corresponding near-isogenic lines (NILs) with yellow and brown seeds (Y-NIL and B-NIL, respectively) and conducted an RNA-seq experiment for the seed coats of the two NILs at different seed developmental stages. Comparative transcriptomic analysis between Y-NIL and B-NIL revealed the core genes involved in the major biological pathways closely related to seed coat pigmentation. In addition, three TF genes located within the *Bnsc-9a* QTL region might function in the seed coat pigmentation.

## Materials and methods

### Plant materials

**Figure 2:**
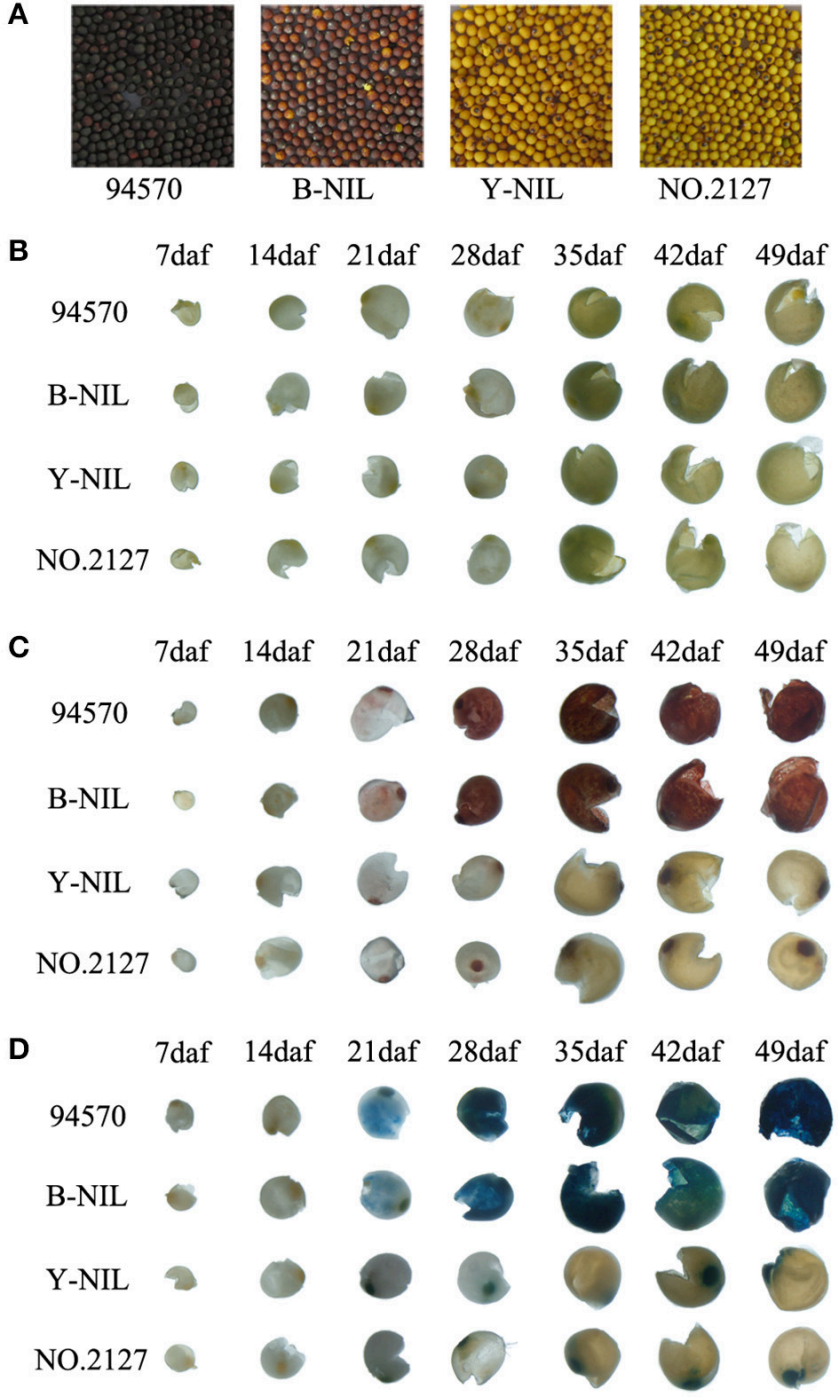
Chemical staining of seed coats from the black-seeded line (94570), brown-seeded near isogenic line B-NIL, and yellow-seeded lines No. 2127-17 and Y-NIL during seed development. **(A)** Seeds from the four lines. **(B–D)** Seed coats before staining **(B)**, after vanillin staining **(C)**, and after DAMACA staining **(D)**. Daf, days after flowering.

Yellow- and brown-seeded NILs (Y-NIL and B-NIL) derived from successive backcrossing using 94570 (black seed) as a donor and No. 2127-17 (yellow seed) as a recurrent parent (Zhang et al., 2011) were grown in the same experimental plot at Huazhong Agriculture University, Wuhan, China. No. 2127-17 was a resynthesized *B. napus* line with double-high quality, while 94570 was a double-low *B. napus* breeding line. The seed fatty acid composition and content were little different between No. 2127-17, B-NIL, and Y-NIL by gas chromatography (GC) analysis. Both Y-NIL and 94570 accumulated significantly higher (~4–8%) seed oil than No. 2127-17 and B-NIL (Supplementary Table 1). Seed coat color was distinct and easy to judge by eye after harvesting (Figure 2A). Before sampling, the seed coat color of each plant was presumed by the two SSR molecular markers, LSR1 and LSR8 (unpublished). Individual flowers of the main raceme and primary branches were tagged on the day of flowering. Tagged developing seeds from four lines were used for comparison analysis at seven developmental stages, namely 7, 14, 21, 28, 35, 42, and 49 days after flowering (daf).

### Vanillin and DMACA staining

The vanillin and DMACA assays are both specific for the detection of (epi)-flavan-3-ols and PAs (Hummer and Schreier, 2008). Two sets of samples that consisted of four seeds and dissected seed sections (seed coats and embryos) from the four rapeseed lines were used for vanillin and DMACA tests, respectively, according to Jiang et al (2013) with some modifications. One set of samples were incubated in a solution of 1.0% (w/v) vanillin in 5 N HCl for 30 min at room temperature. Another set of samples was incubated in a freshly prepared solution of 2% DMACA dissolved in a 6 N HCl/95% ethanol mixture (1:1, v/v) at room temperature for 30 min and then washed for 15 min with water. Samples were observed and photographed under a low-power stereomicroscope (SMZ-U, Nikon, Tokyo, Japan).

### RNA extraction and RNA sequencing

Fresh seed coats were detached manually from the seeds of three individual plants for each NIL at five developmental stages (14–42 daf), and immediately frozen in liquid nitrogen and stored at −80°C for total RNA isolation.

Total RNA was extracted according to the instructions of the RNAprep Pure Plant Kit (TIANGEN, Inc., China). RNA quality (purity and integrity) was monitored by running samples in a 1.0% agarose gels and with an Agilent 2100 Bioanalyzer (Agilent Technologies, Inc.). RNA was quantified using a NanoDrop spectrophotometer (Thermo Fisher Scientific, Inc.). The cDNA library for RNA-seq was prepared using Illumina's TruSeq RNA Sample Preparation Kit according to the manufacturer's instructions (Illumina, Inc.). In total, 30 libraries were sequenced using a paired-end read protocol on the Illumina Hiseq™2500 sequencing platform at the National Key Laboratory for Crop Genetic Improvement, Huazhong Agricultural University, Wuhan, China. The raw sequence data was deposited in the NCBI Sequence Read Archive (Accession No. SRP107899).

### Data processing and read mapping

The quality control (QC) for the raw reads was assessed using the NGS QC Toolkit v2.3.3 (Patel and Jain, 2012) at default parameters for removing the reads containing adapters and poly-Ns, as well as the low-quality reads to obtain the clean reads. Then the clean reads from each library were aligned against the *B. napus* var. Darmor-*bzh* genome (Chalhoub et al., 2014) using HISAT2 (https://ccb.jhu.edu/software/hisat2/index.shtml; Kim et al, 2015) with default settings. Only uniquely mapped reads were considered for further analysis.

### Identification and analysis of DEGs

Gene expression levels (feature counts) were measured using the featureCounts program (Liao et al., 2014). Genes with <10 counts from the sum of three biological replicates in all the samples were considered as unexpressed genes. Differentially expressed gene (DEG) analysis was performed with the DESeq2 R package (Love et al., 2014). Genes with a false discovery rate (FDR) ≤0.05 and an absolute value of log_2_ fold change ≥1 between NILs at each stage were defined as DEGs. Hierarchical clustering of DEGs at five stages was performed using R based on the normalized feature counts [log_2_ (1 + counts)] from each sample. DEGs also were clustered using the K-means method with Genesis (Sturn et al., 2002) based on their feature counts.

The gene ontology (GO) terms of entire sets of *B. napus* genes were annotated as described by Wu et al. (2016). The website tool WEGO (http://wego.genomics.org.cn/cgi-bin/wego/index.pl; Jia et al., 2006) was employed to produce GO functional classification and illustrate the distribution of gene classification. GO enrichment analysis of the DEGs compared to the reference genome, was performed using Blast2GO (Conesa et al., 2005) with an FDR≤0.01. The redundancies of significantly enriched GO terms were reduced using REVIGO (http://revigo.irb.hr/; Supek et al., 2011) with a cut-off value of *C* = 0.4. Kyoto Encyclopedia of Genes and Genomes (KEGG) pathway enrichment analysis was performed using KOBAS 2.0 (http://kobas.cbi.pku.edu.cn; Xie et al., 2011) with a corrected *p* ≤ 0.05 based on *B. napus* CDS BLASTN results from an alignment against the entire genome. Furthermore, homologs of *A. thaliana* genes in the *B. napus* genome were identified using the BlastP program with an *E* ≤ 1E-05, identity ≥50%, and coverage ≥50% (Wu et al., 2016).

### qRT-PCR validation

Twenty-three DEGs were chosen for validation of the RNA-Seq data using the qRT-PCR (quantitative real-time PCR) technique. Four genes [*BnaC08g12720D* (*UBC9*), *BnaA10g06670D* (*UBC10*), *BnaA09g14410D* (*PP2A-1*), *BnaC02g00690D* (*ACT7*)] were used as internal controls (Wu et al., 2016). Primers were designed based on the reference genome sequences using Primer Premier 5.0 and are presented in Supplementary File 1. First-strand cDNA was synthesized from 2 μg RNA per sample (the same samples as for RNA-Seq) using RevertAid First Strand cDNA Synthesis Kit (Thermo Fisher Scientific, Inc.) according to the manufacturer's protocol. The qRT-PCR was performed using SYBR® Green Realtime PCR Master Mix (TOYOBO) in a Bio-Rad CFX-96 RealTime PCR System (Bio-Rad). Relative expression levels were calculated with LINREG (Ramakers et al., 2003). Scatter plot and Pearson correlation coefficients were calculated using an R package with log_2_ fold changes in qRT-PCR and RNA-Seq data.

## Results

### Detection of PAs in the seed coats

Vanillin and DMACA staining showed that PAs did not accumulate in the seed coats of all four lines at both 7 and 14 daf (Figures 2C,D). Red coloration with vanillin and blue coloration with DMACA started at 21 daf in the black and brown seed coats of 94570 and B-NIL, respectively, and the color gradually become darker during development (Figures 2B–D). However, staining did not occur in the yellow seed coats at any stages, except for the stained hilum. These results illustrated that PA content in yellow seed coats was much lower than that in black/brown seed coats. No differences were observed in the undamaged seeds or embryos of the four lines before or after staining (Supplementary Figure 1), indicating the differences in PAs between black/brown and yellow seeds distinctively occurred in the inner endothelium. These results are similar to previous studies that showed that PA content differs between black/brown and yellow seed coats of *B. napus* and *A. thaliana* (Dixon et al., 2005; Auger et al., 2010; Jiang et al, 2013; Qu et al., 2013).

### Transcriptome sequencing and read mapping

To investigate the molecular changes that take place in yellow seed coats of *B. napus*, transcriptome sequencing was performed for 30 libraries prepared from seed coats of the two NILs at five developmental stages (14–42 daf; Supplementary Table 2). After quality control and read mapping, ~28.9–67.4 million uniquely mapped reads across all of the samples were retained for further analysis. Based on the gene expression level calculated by featureCounts (Liao et al., 2014), ~55,562–61,058 genes were expressed in the different seed coats at the five developmental stages (Supplementary Figure 2). We found a close overlap in genes expressed between the NILs; 48,430 common genes were expressed at each stage in both NILs. Based on rlog-transformed counts calculated by the *rlog* function in the DESeq2 package, Pearson correlation coefficients between each pair of biological replicates at each stage for each NIL were high (*R* > 0.92 in most cases; Supplementary Figure 3). These results showed that the sequencing data used in the present study was highly reliable.

### Identification of differentially expressed genes

**Figure 3:**
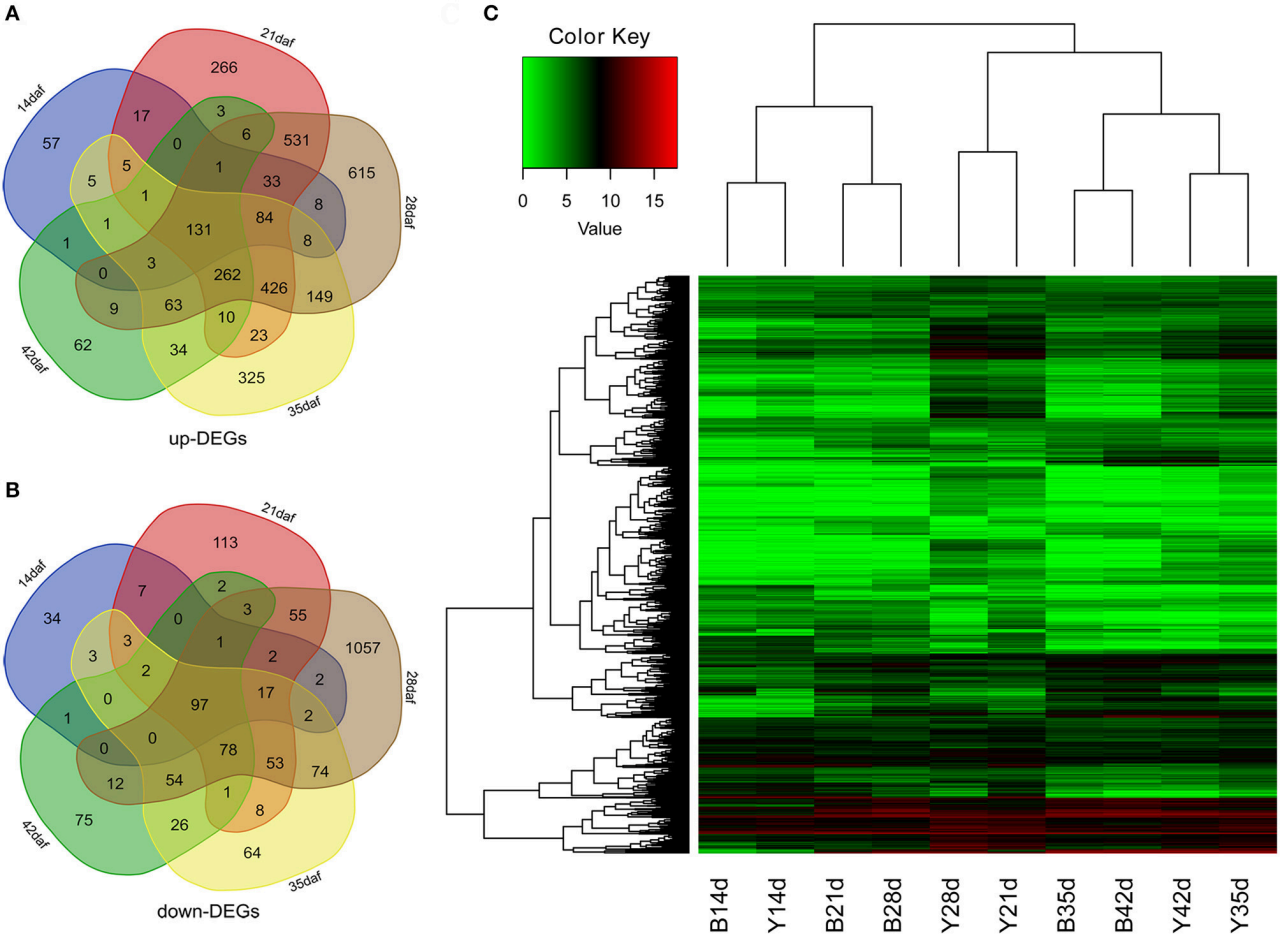
Expression analyses of DEGs in yellow and brown seed coats during seed development. **(A,B)** Venn diagrams of up-DEGs **(A)** and down-DEGs **(B)** at 14, 21, 28, 35, and 42 daf, respectively. **(C)** A heatmap showing the expression profiles of DEGs according to the normalized feature counts [log_2_ (1 + counts)]. The red bands indicate high gene expression and the green bands indicate low gene expression.

To obtain the gene expression changes in the seed coat of Y-NIL, we used the DESeq2 to examine the DEGs between Y-NIL and B-NIL at each stage (14, 21, 28, 35, and 42 daf), respectively. A total of 4,974 DEGs were identified during seed development, of which 11 genes were up- or down-regulated in Y-NIL at different stages (Supplementary File 2). The up-regulated genes (up-DEGs) dramatically outnumbered the down-regulated genes (down-DEGs) at each stage, namely 355:171, 1,799:442, 2,329:1,507, 1,530:482, 587:352, respectively (Figures 3A,B). One hundred and thirty-one up-DEGs and 97 down-DEGs were detected in all five stages. Hierarchical cluster analysis of all the DEGs was performed to show the overall gene expression pattern of the DEGs (Figure 3C).

All DEGs were also divided into 16 clusters based on their expression patterns at seed developmental stages using Genesis (Sturn et al., 2002; Supplementary Figure 4). The DEGs in most clusters showed a similar developmental pattern between B-NIL and Y-NIL, except for clusters 3, 4, 8, and 14. Moreover, expression of the DEGs in some clusters were the most significantly different from 21 daf onward, including the up-DEGs in clusters 3, 8,13, and 14, as well as the down-DEGs in clusters 7, 10, 11, and 12, which indicated that the stage between 14 and 21 daf is the key stage for seed coat coloration.

### GO and KEGG pathway enrichment analyses of DEGs

**Figure 4:**
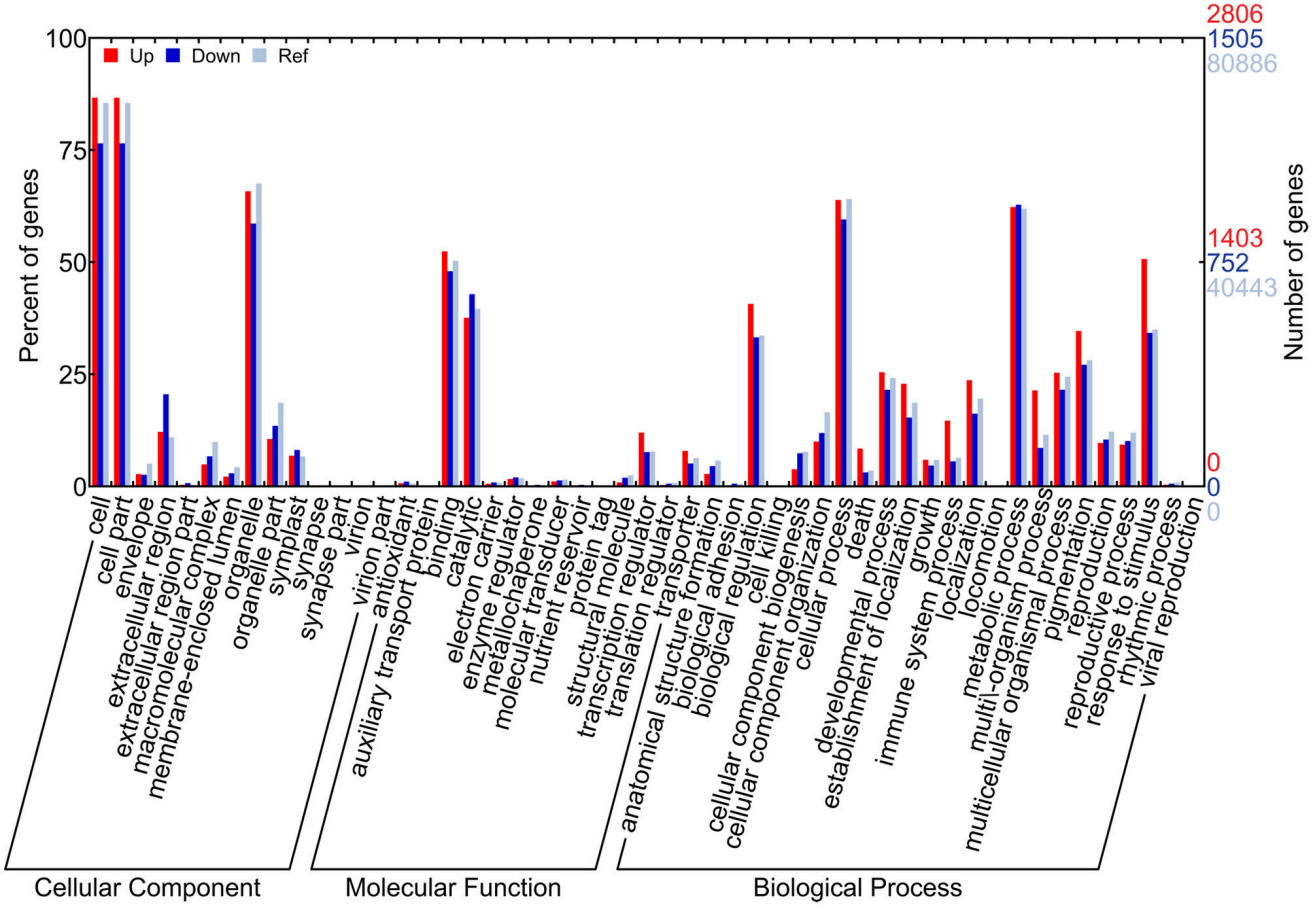
GO term classification for DEGs by WEGO. The x-axis indicates the sub-categories, the left y-axis indicates the percentage of a sub-category of genes in that category, and the right y-axis indicates the number of genes in a sub-category.

A total of 4,301 DEGs (2,806 for up-DEGs, 1,505 for down-DEGs, including 11 up-/down-DEGs) were annotated in at least one GO term and assigned to 47 main GO terms under three GO categories: biological process, cellular component, and molecular function (Figure 4). In the GO enrichment analysis, the most representative term in biological process for up-DEGs was “response to stimulus” (1,473 DEGs), whereas the representative terms for down-DEGs were “phenylpropanoid metabolic process” (81 DEGs) and “flavonoid metabolic process” (85 DEGs; Table 1). The molecular function term “nucleic acid binding transcription factor activity” was highly enriched in up-DEGs (Supplementary File 3). By contrast, the enriched molecular function terms for down-DEGs were almost exclusively associated with oxidoreductase activity. Interestingly, no GO terms were enriched within the down-DEGs at 14 daf, and phenylpropanoid and flavonoid metabolism processes were overrepresented after 21 daf (Supplementary File 4).

**Table 1:** Top 10 of enriched GO terms for DEGs in yellow and brown seed coats.

	**GO ID**	**GO Term**	**GO Type^a^**	**log10 *p*-value**	**No. of DEGs**
Up-DEGs	GO:0010243	Response to organonitrogen compound	P	−154.8239	411
	GO:0009719	Response to endogenous stimulus	P	−85.7696	713
	GO:0009620	Response to fungus	P	−67.6778	317
	GO:0050896	Response to stimulus	P	−59.6576	1473
	GO:0002376	Immune system process	P	−53.9208	411
	GO:0045730	Respiratory burst	P	−53.6198	130
	GO:0002679	Respiratory burst involved in defense response	P	−53.6198	130
	GO:0009693	ethylene Biosynthetic process	P	−48.3768	112
	GO:1900673	Olefin metabolic process	P	−48.3768	112
	GO:0023052	Signaling	P	−47.3665	602
Down-DEGs	GO:0005576	Extracellular region	C	−24.0706	309
	GO:0009698	Phenylpropanoid metabolic process	P	−23.8539	81
	GO:0009813	Flavonoid biosynthetic process	P	−20.585	80
	GO:0009812	Flavonoid metabolic process	P	−20.2757	85
	GO:0019748	Secondary metabolic process	P	−13.3979	132
	GO:0010224	Response to UV-B	P	−9.4949	39
	GO:0009611	Response to wounding	P	−5.2147	73
	GO:0045548	Phenylalanine ammonia-lyase activity	F	−5.9208	7
	GO:0016710	Trans-cinnamate 4-monooxygenase activity	F	−5.4437	6
	GO:0016872	Intramolecular lyase activity	F	−5.0809	10

a *P, biological process; C, cellular component; F, molecular function*.

KEGG pathway analysis showed that 467 up-DEGs and 319 down-DEGs were mapped to 101 and 103 pathways, respectively (Supplementary File 5). Seven pathways were significantly enriched with 181 up-DEGs, including “plant–pathogen interaction,” “plant hormone signal transduction,” “fatty acid elongation,” “protein processing in endoplasmic reticulum,” “zeatin biosynthesis,” “cutin, suberine, and wax biosynthesis,” and “ABC transporters” (Table 2). Most of the up-DEGs involved in these pathways are assigned in the GO term “response to stimulus” (Supplementary Files 3, 5). In contrast, “flavonoid biosynthesis,” “phenylpropanoid biosynthesis,” “phenylalanine metabolism,” “degradation of aromatic compounds,” “biosynthesis of secondary metabolites,” and “propanoate metabolism,” were the top six pathways represented by 128 down-DEGs (Table 2). The pathway analysis provided a better functional insight into the DEGs and validated the GO enrichment analysis in which DEGs were predominantly correlated to secondary metabolism and response to stimulus.

**Table 2:** Enriched KEGG pathways for DEGs in yellow and brown seed coats.

	**Pathway**	**Pathway ID^a^**	**No. of DEGs**	**No. of background genes**	**Corrected *P*-value**
Up-DEGs	Plant–pathogen interaction	ath04626	59	664	4.1433*E*−14
	Plant hormone signal transduction	ath04075	52	1107	0.000262056
	Fatty acid elongation	ath00062	11	108	0.002428641
	Protein processing in endoplasmic reticulum	ath04141	36	801	0.00817563
	Zeatin biosynthesis	ath00908	8	72	0.009286465
	Cutin, suberine and wax biosynthesis	ath00073	9	98	0.014826493
	ABC transporters	ath02010	8	86	0.024094666
Down-DEGs	Flavonoid biosynthesis	ath00941	33	93	1.82616*E*−24
	Phenylpropanoid biosynthesis	ath00940	35	578	6.09979*E*−06
	Phenylalanine metabolism	ath00360	17	175	3.83049*E*−05
	Degradation of aromatic compounds	ath01220	7	34	0.000805237
	Biosynthesis of secondary metabolites	ath01110	123	4138	0.001479341
	Propanoate metabolism	ath00640	8	111	0.047040149

a *Pathway from Arabidopsis thaliana database*.

### Comprehensive analysis of the genes involved in flavonoid biosynthesis

The results of GO and pathway analyses made clear that the phenylpropanoid and flavonoid biosynthesis were significantly altered after 14 daf in the seed coats of Y-NIL compared to B-NIL (Supplementary File 6).

In the general phenylpropanoid pathway (Saito et al., 2013), the expression of three genes was suppressed to different extents during seed development in Y-NIL. Four copies of *PAL2* gene were significantly down-regulated at 28–42 daf, but only one copy of *PAL1* (*BnaC04g08190D*) was down-regulated at 21–35 daf and another copy of *PAL1* (*BnaA05g07370D*) was expressed at low levels in the seed coats. Down-regulation of *C4H* were found in six homologs at 21–35 daf. Among 4CL homologs, all two copies of *4CL3*, which may be important for flavonoid biosynthesis, were down-regulated after 14daf; however, the expression level of one *4CL1* homolog (*BnaA05g15310D*) that is likely responsible for lignin biosynthesis slightly decreased at 28 and 35 daf. Conversely, one *4CL5* homolog (*BnaA05g36600D*) was highly expressed at 35 daf in Y-NIL. The expression of some copies of other genes involved in the phenylpropanoid derivative pathway was also changed in Y-NIL (Supplementary Figure 5), such as, the up-DEGs, *BnaC02g46610D* (*CCR2*), *BnaA02g36250D* (*CCR2*), *BnaA06g40660D* (*CAD9*), *BnaA01g15400D* (*CCoAOMT7*), and *BnaC01g18290D* (*CCoAOMT7*), as well as the down-DEGs, *BnaA01g18180D* (*HCT*) and *BnaC02g13760D* (*OMT1*). However, the lignin biosynthesis seemed unaffected by these expression changes in yellow seed coats since other copies of these genes involved in lignin synthesis (Fraser and Chapple, 2011; Yu, 2013) showed higher expressed levels in the seed coats, and lignin biosynthesis or cell wall metabolism were not found to be enriched in GO and KEGG analyses.

In flavonoid biosynthesis, all the structural genes apart from *F3*′*H* showed significant down-regulation during the four developmental stages except 14 daf, including six copies of *CHS*, three copies of *CHI*, three copies of *F3H, DFR, ANS*, and *ANR* genes (Figure 1 and Supplementary File 6). However, the expression levels of two other copies of *CHI* (*BnaA09g34850D* and *BnaAnng08210D*) were increased at 21 and 28 daf and were far less than those of the down-regulated *CHI* copies, implying that the two up-regulated *CHI* homologs may not be the main copies expressed in the *B. napus* seed coat. For *F3*′*H*, only one copy (*BnaC09g47980D*) was identified as a down-DEG at 28 daf. The expression level of another *F3*′*H* copy (*BnaA10g23330D*) in Y-NIL was less than twice that in B-NIL at 28 and 35 daf, suggesting that *F3*′*H* is not the main cause of testa color differences. The expression of genes encoding the key transporters, *TT12, AHA10*, and *GST26*, were also repressed at different stages in Y-NIL, similar to what was detected in the structural genes. One homolog of *TT12* (*BnaC06g17050D*) showed significant down-regulation during the mid-to-late stages but not at 21 daf. *AHA10* and two copies of *TT19* with lower transcript abundance were down-regulated at 21–42 daf, whereas the expression level of the highly transcribed *TT19* homolog (*BnaA10g17440D*) had not significant difference at mature stages (35 and 42 daf). Yet surprisingly, *LAC15* and *TT15*, which are the downstream genes of the flavonoid pathway, did not show significantly different expression levels, suggesting that the accumulation of epicatechin was blocked in Y-NIL. For flavonol biosynthesis, no difference (FDR > 0.05) was observed in the expression of *FLS* and only one out of 16 copies of *OMT1* were down-regulated at mature stages. Furthermore, whether the EBGs or the LBGs, represented by *CHS, CHI, F3H, DFR, ANS, ANR, TT12, AHA10*, and *TT19*, were primarily grouped into clusters 11 and 12 by the K-mean method, and had similar expression profiles in the developing seed coat (Supplementary File 6), proposing that these genes might be regulated by an upstream regulator affecting the seed coat color formation in *B. napus*.

For reported regulators related to flavonoid biosynthesis, the expression of a few genes was also changed at different developmental stages in Y-NIL (Supplementary File 6). Up-DEGs included one *TT16*, one *MYB75*, one *EGL3*, one *MYC1*, one *MYC2*, and one *LWD1* (*AN11*) homolog. Down-DEGs contained one *TT16*, three *MYB3*, one *MYB4*, four *CPC*, two *TT8*, and two *TT1* homologs. The down-regulated *TT16* copy showed significant difference at 28–42 daf, but another *TT16* copy showed only a slight up-regulation at 28 daf. For the MYB genes, the expression levels of *MYB75* and *MYB90* involved in anthocyanin biosynthesis were extremely low in the seed coats of both NILs. Some copies of *MYB3, MYB4*, and *CPC* were detected with decreases in transcriptional levels in Y-NIL, although the coding proteins of these three genes act as repressors in phenylpropanoid and anthocyanin biosynthesis in *A. thaliana* (Dubos et al., 2010). As a bHLH protein that is a key member of MBW complex, one copy of *TT8* (*BnaA09g22810D*) was down-regulated by 2-fold at 21 and 28 daf, and another copy (*BnaC09g24870D*) only at 28 daf. In our previous mapping studies using NILs (unpublished), we found that *TT8* was not a candidate gene for the QTL *Bnsc-9a* (Zhang Y. et al., 2009; Zhang et al., 2011; Li X. et al., 2012). *BnaC09g11380D* (*EGL3*) showed significant up-regulation only at 35 and 42 daf. All two copies of *TT1* were down-regulated by 4-fold at 21–42 daf, indicating that BnTT1 plays a key role in the regulation of flavonoid biosynthesis in our conditions. However, silencing of the *BnTT1* genes in black-seeded *B. napus* cultivar ZS10 increased the *LAC15* and *UGT*s transcript levels and produced a brown seed coat (Lian et al., 2016), which were different from the results that *LAC15* expression was not changed in Y-NIL with light yellow seed coats in the present study. These results indicated that some unreported regulatory genes might be involved in flavonoid pathway in *B. napus*.

### Identification of differentially expressed TF genes

To determine the potential candidate genes for the unreported regulators, we identified 474 TF genes belonging to 44 families in 4,974 DEGs based on *B. napus* TFs from the Plant and Transcription Factor Database (PlantTFDB, http://planttfdb.cbi.pku.edu.cn/; Jin et al., 2017; Table 3 and Supplementary File 2). Among 322 up-regulated TF genes, the most abundance TF families were ERF, WRKY, NAC, C2H2, bHLH, MYB, and MYB-related families. These TFs have already been reported to modulate genes involved in plant hormone response, biotic or abiotic stress response and plant growth (Alves et al., 2014). However, *M-type-MADS, MYB, Dof*, *C2H2, MYB*-related, and *HD-ZIP* gene families were overrepresented in the 152 down-regulated TF genes, and are mainly involved in plant growth and development. These results corresponded with the functional analysis of the DEGs, suggesting that these TFs could play important roles in seed coat development and pigmentation. Up-regulated TF transcripts were differentially expressed at one or more of the developmental stages, and had more extreme expression differences than the down-regulated TF genes. Most down-regulated TF genes were differentially expressed at 28 daf, such as, those from MADS family, which are key regulators of plant development processes (Parenicová et al., 2003).

**Table 3:** TF families of DEGs in yellow and brown seed coats.

**TF family**	**Gene number**	
	**Up-regulated**	**Down-regulated**
ERF	65	2
C2H2	36	13
WRKY	41	2
NAC	40	2
MYB	14	18
M-type_MADS	5	21
bHLH	20	4
MYB_related	11	10
Dof	2	14
HD-ZIP	5	7
C3H	7	4
LBD	5	6
bZIP	5	5
MIKC_MADS	3	5
ARR-B	1	6
B3	3	4
GRAS	7	ND
TCP	5	2
AP2	5	1
G2-like	2	4
GATA	3	3
HSF	5	1
Trihelix	5	1
RAV	4	1
YABBY	ND	4
ZF-HD	4	ND
BBR-BPC	2	1
CO-like	3	ND
SBP	3	ND
TALE	ND	3
CAMTA	1	1
DBB	1	1
GeBP	1	1
NF-X1	2	ND
NF-YA	2	ND
ARF	1	ND
BES1	ND	1
CPP	1	ND
HB-other	ND	1
NF-YB	ND	1
Nin-like	ND	1
S1Fa-like	1	ND
WOX	ND	1

*ND, not detected*.

In addition to known TF genes in flavonoid synthesis (*TT1, TT8, TT16, MYB3, MYB4*, and *CPC*), seven TF genes had decreased expression levels in yellow seed coats at all stages or after 14 daf (Supplementary File 6). These seven TFs included one CAMTA, one homeodomain leucine zipper class I (HD-Zip I) protein, two MYB-related, one bHLH, and two C3H proteins. The homologs in *A. thaliana* of three TF genes are annotated to be involved in hormone responses and defense responses (Himmelbach et al., 2002; Yanhui et al., 2006; Nie et al, 2012), including *BnaA09g42730D* (encoding CAMTA3/ SIGNAL RESPONSIVE 1/SR1), *BnaA09g42630D* (encoding HOMEOBOX PROTEIN 6/HB6, a HD-Zip I protein), and *BnaA09g44970D* (homolog of *AT1G17520* encoding a MYB-related protein). However, the function of the homologs of the other four TF genes are still unknown in *A. thaliana*, including one *MYB*-related gene (*BnaA09g44370D*, homologous to *AT1G19000*), two *C3H* genes (*BnaA09g41200D* and *BnaAnng37140D*, homologous to *AT2G24830*), and one *bHLH* gene (*BnaA09g42390D*, homologous to *AT2G22750*). Moreover, the expression profiles of *BnaA09g42730D* and *BnaA09g42630D* in the seed coat were different from those of the other five TF genes and flavonoid-related genes. We noted that except for *BnaAnng37140D*, all the six TF genes are located within the *Bnsc-9a* QTL region on chromosome A09 according to the alignment results of the QTL linked markers (data not shown). Therefore, the identified TF genes which were co-expressed with flavonoid-related genes in this study are potentially candidate regulatory genes for flavonoid biosynthesis or the downstream genes of flavonoid pathway.

### qRT-PCR validation

**Figure 5:**
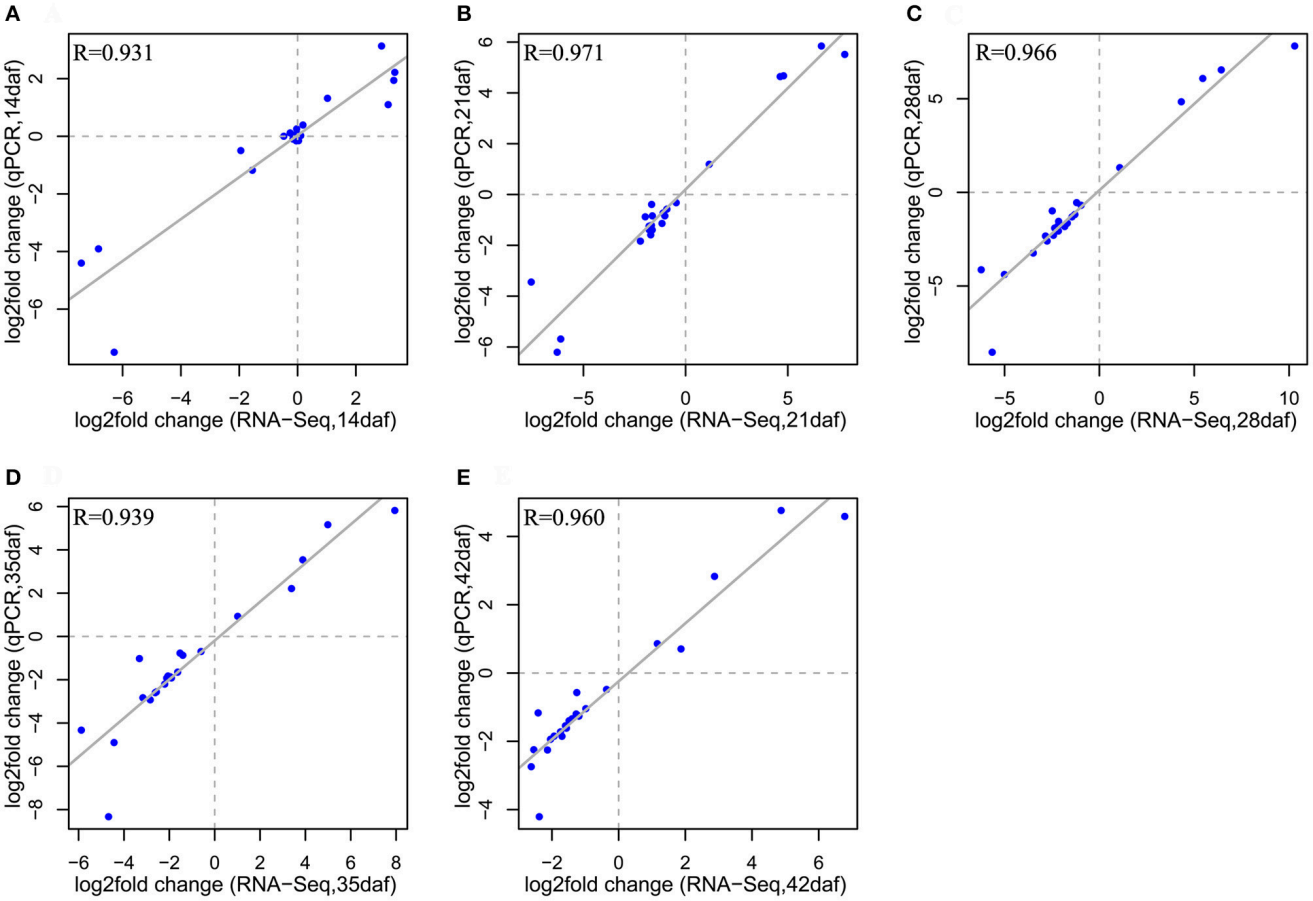
Correlation of gene expression changes from qPCR and RNA-seq methods at each seed developmental stage (14, 21, 28, 35, and 42 daf) **(A–E)**. The log_2_ value of the expression ratio (Y-NIL/B-NIL; y-axis) in the qPCR analysis was plotted against the log_2_ fold change from the RNA-seq data (x-axis).

To verify the RNA-seq data, the expression profiles of 23 DEGs identified in the RNA-seq assays were analyzed by qRT-PCR. These genes included 13 down-DEGs involved in flavonoid biosynthesis and 10 randomly chosen DEGs. All genes involved in flavonoid biosynthesis showed the same expression patterns as in the RNA-seq data, except for *BnaC02g41690D* (*TT16*), which showed no significant difference between NILs in the qRT-PCR analysis (Supplementary Files 6, 7). The expression patterns for *BnaA09g43570D* (*LIP1*), *BnaA09g43050D* (*RER1B*), and *BnaA09g55750D* (*FAP1*) showed little difference between the two techniques, the Pearson correlation coefficients between qRT-PCR data and RNA-seq data at all five developmental stages (*R* = 0.931–0.971; Figure 5) were very high, further confirming the reliability of the RNA-seq data.

## Discussion

Yellow seeds in No. 2127-17 are produced when *D, B*, and *C* loci are homozygous recessive in combination with one dominant allele at the *Y* locus (Zhang Y. et al., 2009). After combining QTL analysis, the QTL *Bnsc-9a* for seed color was detected in linkage group N9 (chromosome A09) and presumably corresponds to the *D* locus (Zhang et al., 2011). To identify the candidate genes for the *D* locus, we investigated the transcriptomes of the yellow (recessive) and brown (dominant) seed coats in the pair of *B. napus* NILs, which carried the major QTL *Bnsc-9a*. Comparative transcriptome analysis between yellow and brown seed coats revealed 4,974 DEGs during seed development, including 3,128 up-DEGs, 1,835 down-DEGs, and 11 up/down-DEGs in the yellow seed coat at different stages (Supplementary File 2). However, the down-DEGs in the *Bnsc-9a* QTL region have been not reported to affect seed coat color formation. Flavonoids including anthocyanins and PAs are the major secondary metabolites that can influence leaf, flower, fruit, and seed color in plants and the flavonoid biosynthesis is controlled by a complex regulatory network with diverse TFs (Xu et al., 2014). Qu et al. (2016b) proposed that some TFs in two expression quantitative trait locus (eQTLs) hotspots, which harbor many *trans*-eQTLs for flavonoid *TT* genes, might be involved in the flavonoid pathway through different regulatory networks; these are represented by *MYB51, bZIP25*, and *MYC1* on chromosome A09. These eQTL hotspots were located around a major QTL for seed coat color on chromosome A09. These genes did not show a transcriptional difference in our conditions. However, six genes encoding putative TFs in the *Bnsc-9a* QTL region were expressed at lower levels at three or four consecutive developmental stages in yellow seed coats, including one *CAMTA3*, one *HB6* (*HD-Zip I*), two *MYB*-related, one *bHLH*, and one *C3H* genes. The expression patterns of *BnaA09g42730D* (*CAMTA3*) and *BnaA09g42630D* (*HB6*) were different from those of the other four TF genes (*MYB*-related, *bHLH*, and *C3H* genes) that resembled those of flavonoid-related genes in the seed coat. CAMTA3 and HB6 mainly be involved in hormone responses and defense responses (Himmelbach et al., 2002; Nie et al, 2012). The C3H proteins were found to have an effective role in stress tolerance, embryogenesis and flower development in *Arabidopsis* (Wang et al., 2008). The reduction in expression level of the two *C3H* genes might be caused by the blocked flavonoid biosynthetic pathway. The MYB and bHLH proteins are involved in seed coat development, cell-wall formation, flavonoid accumulation, and stress responses in plant species; such examples are TT2/MYB123, MYB5, AtMYBL2, AtCPC, TT8, and PhAN1 (Feller et al, 2011). R2R3-MYB proteins, TT2 and MYB5, and bHLH proteins, EGL3 and TT8, respectively, share partially redundancy in TTG1-dependent complexes, and are involved in seed coat differentiation and PA synthesis in the inner seed coat (Gonzalez et al., 2009; Xu et al., 2014). Both AtMYBL2 and AtCPC are R3-type MYBs belonged to MYB-related class in the MYB superfamily (Dubos et al., 2010). Further, AtMYBL2 were shown to directly interact with TT8 to suppress the expression of *DFR* and *TT8* and therefore negatively controls anthocyanin biosynthesis (Matsui et al., 2008). In addition, AtMYBL2 also can inhibit PA biosynthesis and the expression of flavonoid genes in seeds (Dubos et al., 2008). AtCPC is involved in root hair and trichome development, and is also a negative regulator of anthocyanin biosynthesis (Zhu et al., 2009). Additionally, many loci for flavonoid quantitative variation in *A. thaliana* seed did not co-localize with any known flavonoid gene and have yet to be isolated (Routaboul et al., 2012). These findings suggested that the TFs encoded by the three genes within the *Bnsc-9a* QTL region, including two *MYB*-related and one *bHLH* genes, might be novel TFs that potentially participate in the regulatory network of flavonoid biosynthesis to affect the seed coat coloration in *B. napus*.

PAs are one of the end products of the flavonoid biosynthetic pathway in higher plants. It has long been suggested that the seed coat color in *B. napus* is primarily determined by the content of PAs that are constituted by epicatechin units (Yu, 2013). Epicatechin-derived PAs deposition exclusively in the seed coat was blocked after 14 daf in yellow seed coats. The genetic control of pigmentation in the chalaza and micropyle is distinct from that in the endothelium as shown in *Arabidopsis* seeds, inferred by the remaining PAs near the hilum in yellow seed coats (Nesi et al., 2009; Xu et al., 2014). According to transcriptomic analysis, the decrease in the expression level of genes involved in flavonoid biosynthesis such as, *PAL, C4H, 4CL3, CHS, CHI, F3H, DFR, ANS, ANR, TT12, AHA10*, and *TT19*, as well as the regulatory genes *TT1, TT8*, and *TT16*, is consistent with reduced PA content after 14 daf in yellow seed coats. Except for flavonoids, some other phenolic relatives like lignin and melanin, might affect seed coat coloration (Marles and Gruber, 2004; Yu, 2013). Low lignin content is strongly associated with the unpigmented seed coat trait in *Brassica* species (Yu, 2013). However, the expression of genes involved in lignin biosynthesis were slightly changed and lignin biosynthesis is not likely be markedly altered in Y-NIL. Our findings firmly established that the down-regulation of PA-related genes contributes to decreased PA levels, which causes the difference in testa color between brown and yellow seeds. In addition to down-regulated genes, we found that many genes relevant to response to stress were up-regulated in yellow seed coats, including plant–pathogen interaction plant hormone signal transduction and protein processing in endoplasmic reticulum. Flavonoids not only play an essential role in various developmental processes, but also act as scavengers of free radicals (reactive oxygen species, ROS) in protection against stress in plants (Falcone et al, 2012). Significantly reduced PA content in yellow seed coats may result in an increased production of ROS, which act as signals for the activation of stress-response and defense pathways in plants (Baxter et al., 2014), such as, plant–pathogen interaction, plant hormone signal transduction, and protein processing in endoplasmic reticulum. The up-regulation of stress-response genes may be an adaptive strategy by compensating the loss of PAs to ensure seed development and survival in *B. napus*.

Most of PA-related biosynthetic genes showed similar developmental expression patterns in the seed coat, indicating that these genes are functionally related to each other in the flavonoid pathway in *B. napus*, similar to what has described in *Arabidopsis*. Moreover, except for *LAC15* and *TT15*, these genes have similar down-regulation levels in yellow seed coats, suggesting that they might be regulated by the unreported upstream regulator, which might be encoded by the candidate gene for locus *D* in our NILs. The expression of *F3*′*H*, which encodes a cytochrome P450 monooxygenase that converts dihydrokaempferol or kaempferol to dihydroquercetin and quercetin, may be not controlled by the regulatory network of the potential upstream regulators for PA-related biosynthetic genes. F3′H might be not responsible for seed color formation in *B. napus*, since *F3*′*H* was found to show no or limited down-regulation in some yellow-seeded *B. napus* lines (Lian et al., 2016; Qu et al., 2016a). The regulation of LBGs for PAs accumulation (*DFR, ANS, ANR, TT12, AHA10*, and *TT19*) primarily occurs via the TT2–TT8–TTG1 complex and other TFs (TT1, TTG2, and TT16) that act in PAs-accumulation cells of *Arabidopsis* seed (Xu et al., 2014). Among these known TF genes, only *TT1* showed identical expression regulation as the LBGs in Y-NIL. In *Arabidopsis*, the activity of the *TT8* promoter could be regulated by three TFs, TT1, TT16, and TTG2 (Xu et al., 2013). It may be that *TT8* showed decreased expression level only at 21 and 28 daf because of the regulatory link between TT1, TT16, and TT8. *TT1* encodes a WIP zinc finger protein that is required for the endothelium cells to be able to synthesize PAs during seed coat differentiation (Sagasser et al., 2002), and can also interact with TT2 involved in MBW complexes (Appelhagen et al., 2011). Suppression of *BnTT1* genes not only reduced expression of *CHS, DFR, ANS, ANR*, and *TT12* as in *Arabidopsis*, but also that of *CHI, F3H*, and *TT19* in *B. napus* seed (Lian et al., 2016). Lower *CHS* expression level regulated by TT1 may trigger down-regulation of EBGs (*CHI, F3H*, and *F3*′*H*) in Y-NIL after 14 daf. However, the expression level of *BnTT1*s was found to peak at the early stage of seed development (7 daf) in the seed coat, and had no difference between NILs in the qRT-PCR analysis (Supplementary File 7). The regulation of the potential upstream regulators on *TT1* expression might occur during mid-to-late developmental stages. The two *MYB*-related and one *bHLH* genes within the *Bnsc-9a* QTL region, which showed similar expression patterns to the LBGs in PA biosynthesis, might be involved in the upstream regulatory network of PA-related core genes, including the LBGs and the regulatory gene *TT1*. Methylation affects a MYB transcription factor at the *R* locus in soybean, which can regulate *ANS* and might be responsible for variation in the seed coat color (Zabala and Vodkin, 2014). The candidate genes for the *Bnsc-9a* QTL may affect the transcription of three identified potential TFs through epigenetic regulation. Further studies will be needed to determine the functions of these down-regulated TF genes in the QTL region during seed coat development and pigmentation in *B. napus*.

In summary, our study uncovered the transcriptome variance between yellow and brown seed coats during seed development in a pair of *B. napus* NILs using RNA-seq analysis. The down-regulation of genes involved in PA biosynthesis plays a crucial role in the formation of the yellow seed coat in *B. napus* and might be regulated by a yet un-annotated regulator encoded by the candidate of dominant black-seeded gene *D*. Our study identifies novel potential TFs involved in flavonoid biosynthesis and will provide pivotal information for identifying the yellow-seeded candidate genes in *B. napus*. This vast transcriptome dataset will lay the foundation for future functional studies of key candidate genes responsible for seed coat coloration and provide the expression files of seed coat development in *B. napus*.

## Author contributions

MH and JT conceived and designed the experiments. MH, XL, LC, and YZ performed the experiments. KH contributed to bioinformatics analysis and TT performed the qPCR assays. BY, JW, CM, JS, and TF, contributed reagents/materials/analysis tools. MH analyzed the data and wrote the manuscript. JT reviewed the manuscript.

### Conflict of interest statement

The authors declare that the research was conducted in the absence of any commercial or financial relationships that could be construed as a potential conflict of interest.
