# Supplementary material for: Transcriptomic Analysis of Seed Coats in Yellow-Seeded Brassica napus Reveals Novel Genes That Influence Proanthocyanidin Biosynthesis
Source: Front Plant Sci. 2017 Oct 5;8:1674. doi: 10.3389/fpls.2017.01674 (PMC5633857; doi:10.3389/fpls.2017.01674)
Supplement: Supplementary file 8 [file Presentation1.pdf]

Supplementary Material

Transcriptomic Analysis of Seed Coats in Yellow-seeded *Brassica napus* Reveals Novel Genes that Influence Proanthocyanidin Biosynthesis

Meiyan Hong[1], Kaining Hu[1], Tiantian Tian[1], Xia Li[2], Li Chen[3], Yan Zhang[4], Bin Yi[1], Jing Wen[1], Chaozhi Ma[1], Jinxiong Shen[1], Tingdong Fu[1], Jinxing Tu[1*]

*** Correspondence:** Jinxing Tu, tujx@mail.hzau.edu.cn

1 Supplementary Data

Supplementary File 1. Sequences of the primers used for qRT-PCR analysis.

Supplementary File 2. DEGs in yellow and brown seed coats during seed development (14-42 days after flowering (daf)).

Supplementary File 3. Enriched GO terms of DEGs during seed development using Blast2GO.

Supplementary File 4. Statistics of the enriched GO terms of DEGs at each seed developmental stage.

Supplementary File 5. KEGG pathways of DEGs during seed development.

Supplementary File 6. Expression levels of DEGs involved in phenylpropanoid and flavonoid pathways and other DEGs identified in the RNA-seq analysis.

Supplementary File 7. Relative expression levels of DEGs in seed coats from NILs (B-NIL and Y-NIL) and parents (94570 and No. 2127-17) during seed development by qRT-PCR analysis.

2 Supplementary Figures and Tables

2.1 Supplementary Figures

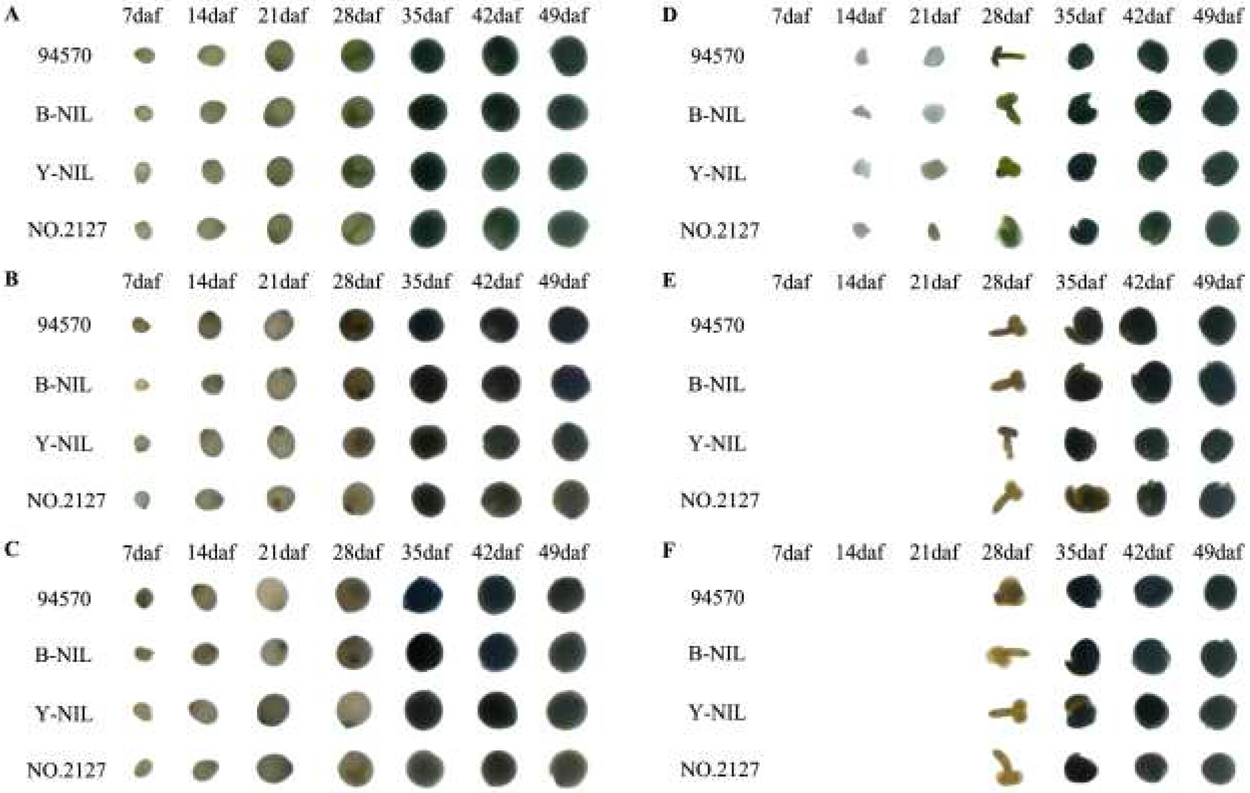

Supplementary Figure 1. Chemical stainings of seeds and embryos from four *B. napus* lines at 7, 14, 21, 28, 35, 42, and 49 daf. **(A-C)** Seeds before staining **(A)**, after vanillin staining **(B)**, and after DMACA staining **(C)**. **(D-F)** Embryos before staining **(D)**, after vanillin staining **(E)**, and after DMACA staining **(F)**. daf, days after flowering.

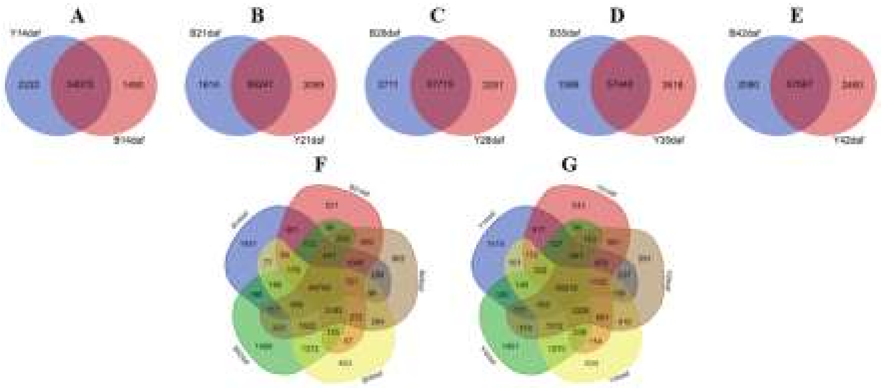

Supplementary Figure 2. Number of expressed genes (EGs) in B-NIL and Y-NIL during seed development. **(A-E)** Venn diagrams of expressed genes in B-NIL and Y-NIL at each developmental stage (14, 21, 28, 35, and 42 daf). **(F, G)** Venn diagrams of expressed genes of five stages for B-NIL **(F)** and Y-NIL **(G)**, respectively. EGs, Genes with more than 10 counts from the sum of three replicates in each sample.

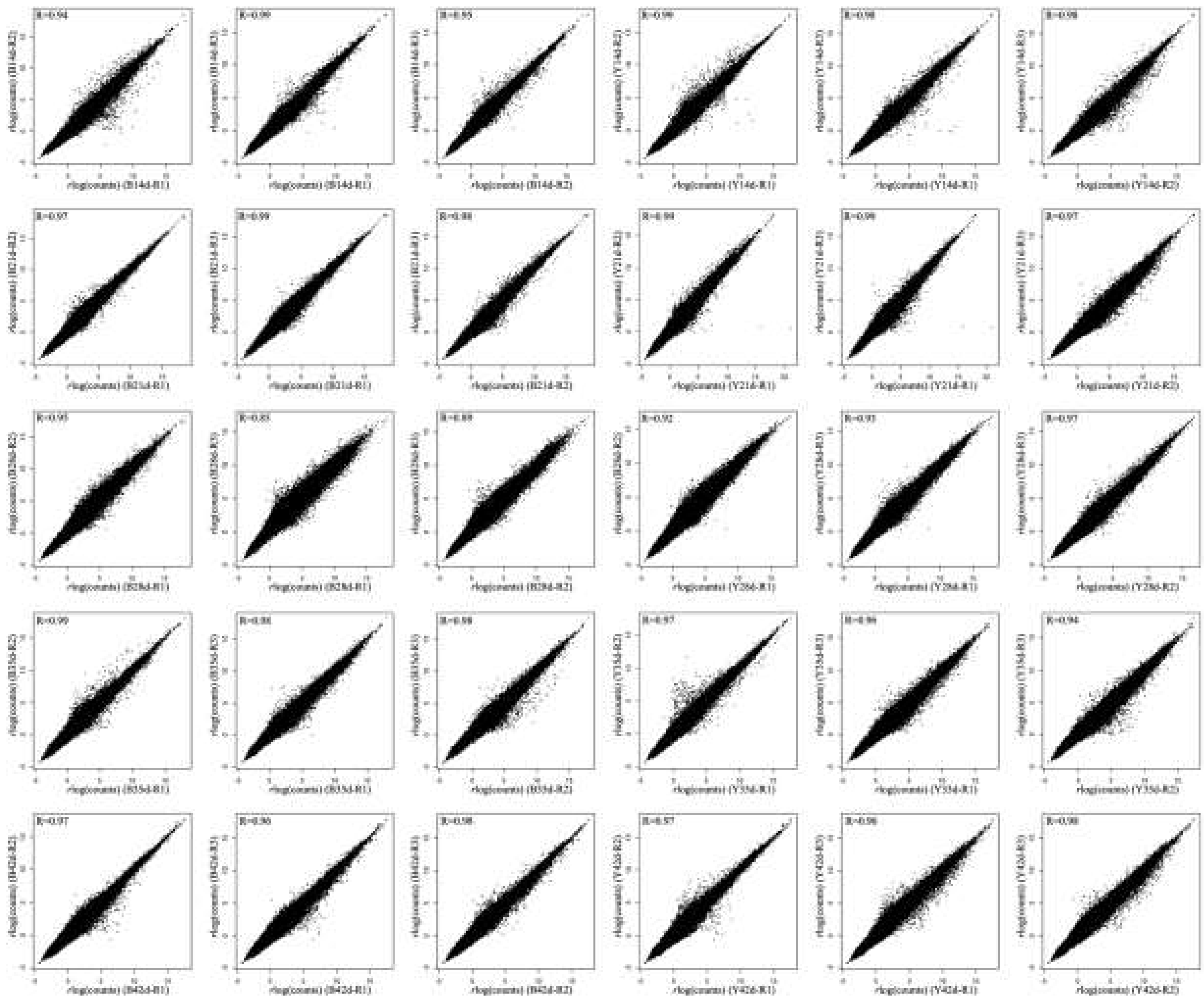

Supplementary Figure 3. Pearson correlation coefficients between each pair of biological replicates at different developmental stages for both B-NIL and Y-NIL based on the rlog-transformed feature counts.

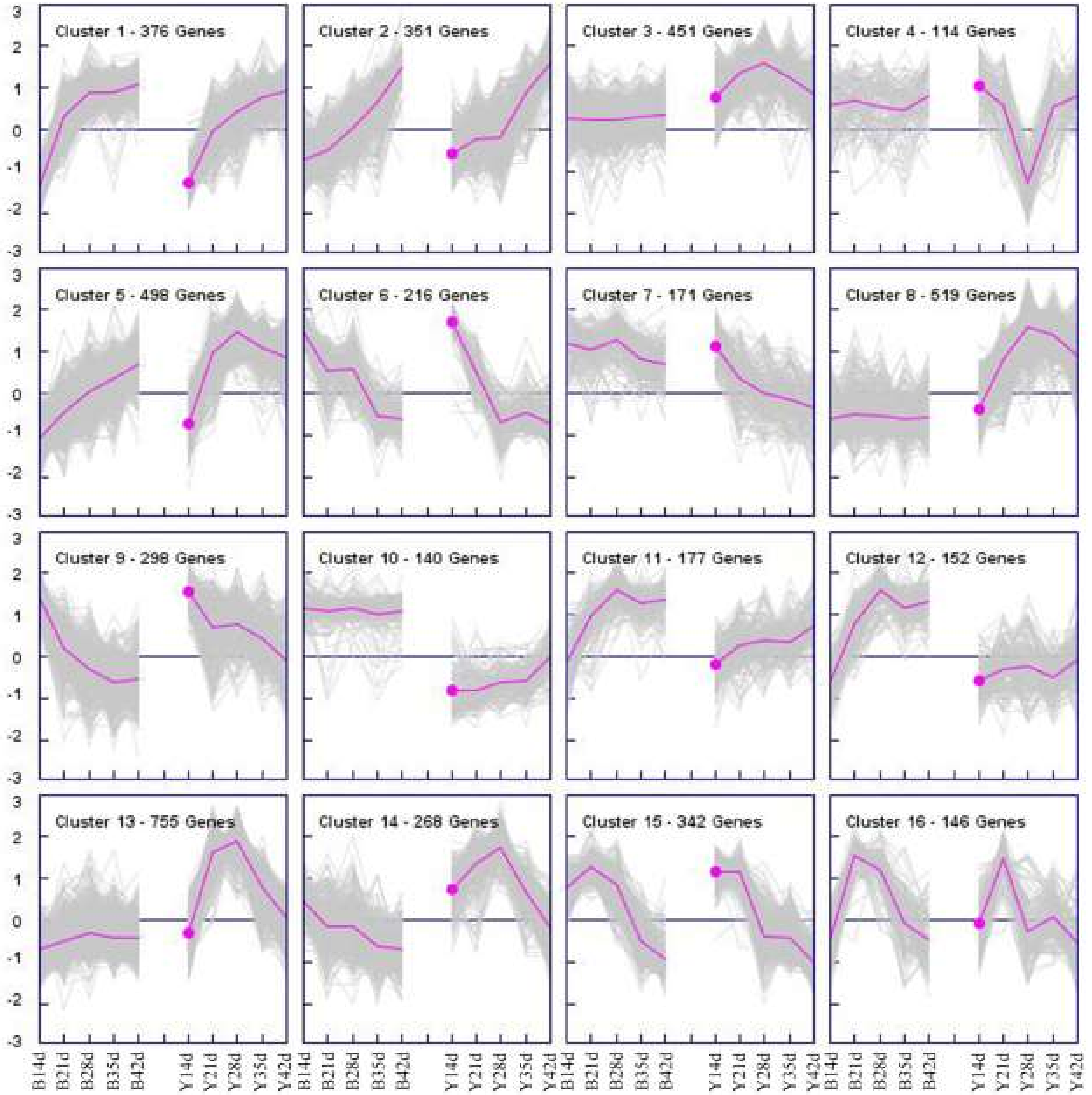

Supplementary Figure 4. Cluster analysis of all 4,974 DEGs. DEGs were classified to 16 clusters by K-means clustering method based on normalized feature counts (y-axis) with Genesis.

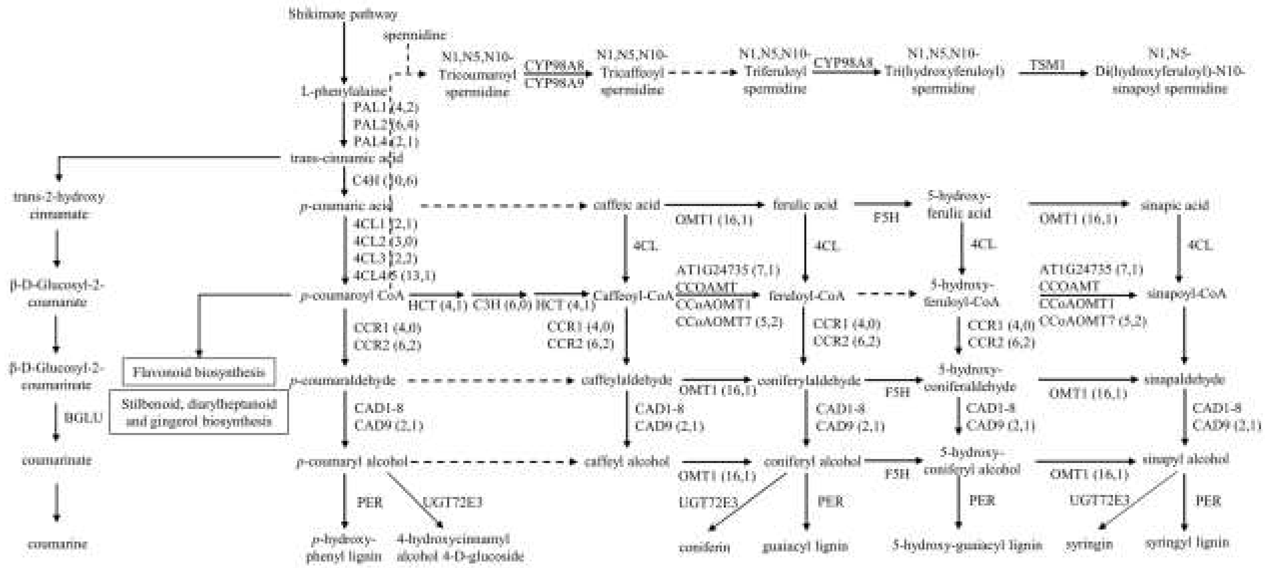

Supplementary Figure 5. Phenylpropanoid pathway for lignin biosynthesis containing DEGs in *B. napus* seed coats (adapted from Yu (2013)). The copy number of genes from the whole genome and from the DEGs in the present study are listed in parentheses, respectively. PAL, phenylalanine ammonia-lyase; C4H, cinnamate 4-hydroxylase; 4CL, 4-coumarate:CoA ligase; HCT, hydroxycinnamoyl-coenzyme A shikimate: quinate hydroxy-cinnamoyl-transferase; C3′H, 4-coumarate 3′-hydroxylase; CYP98A8, cytochrome P450, Family 98, Subfamily A; OMT1, O-methyltransferase 1; CCOAMT, caffeoyl-CoA 3-o-methyltransferase; CCoAOMT, caffeoyl coenzyme A O-methyltransferase; F5H, ferulate-5-hydroxylase; CCR, cinnamoyl-CoA reductase; CAD, cinnamyl alcohol dehydrogenase; UGT72E3, UDPG: coniferyl alcohol glucosyltransferase; BGLU, beta glucosidase; PER, Peroxidase.

2.2 Supplementary Table

Supplementary Table 1. Fatty acid (FA) profile (% composition) in mature seeds of four B. napus lines. FA composition content is presented as percentage content as proportion of total FA from GC analysis. FA content is presented as percentage content in dry seeds (% DW). Oil and protein contents are presented as percentage contents in dry seeds (% DW) from NIRS. Values are means ± SE of three biological repeats for parents and five biological repeats for NILs.

	**94570**	**No. 2127-17**	**B-NIL**	**Y-NIL**
Palmitic	5.56 ± 0.001a	4.75 ± 0.002ab	5.64 ± 0.002b	5.16 ± 0.002
Stearic	2.75 ± 0.001a	1.73 ± 0.001ab	2.66 ± 0.002b	2.29 ± 0.002
Oleic	63.23 ± 0.007a	18.44 ± 0.003a	19.98 ± 0.005a	20.28 ± 0.003a
Linoleic	18.26 ± 0.003	19.68 ± 0.003	20.08 ± 0.006a	18.36 ± 0.003a
Linolenic	8.7 ± 0.002a	11.87 ± 0.004a	10.92 ± 0.003a	11.44 ± 0.002a
Eicosenoic	ND	12.39 ± 0.004a	13.23 ± 0.003	13.86 ± 0.003a
Erucic	ND	27 ± 0.006	25.48 ± 0.005	27.01 ± 0.003
FA content (% DW)	44.59 ± 0.01ab	39.26 ± 0.00ac	35.91 ± 0.00bd	43.60 ± 0.00cd
Oil content (% DW)	41.60 ± 0.35a	39.21 ± 1.11	37.36 ± 0.52ab	41.50 ± 0.81b
Protein content (% DW)	28.48 ± 0.49	28.21 ± 1.23	28.38 ± 0.54	28.10 ± 1.13

[a] Different lowercase letters indicates significant difference at the $P < 0.05$ in a *t*-test.

Supplementary Table 2. Summary statistics of RNA-seq reads and mapped reads.

Sample NO.[a]	No. of Raw reads	No. of Clean reads	Percent of Mapped reads[b]	Percent of Uniquely mapped reads[c]	Percent of Properly paired reads[b]
B14d-R1	50,034,118	50,023,134	86.59%	78.46%	69.71%
B14d-R2	73,215,200	73,197,044	84.18%	79.30%	65.79%
B14d-R3	43,068,266	43,061,802	86.15%	79.98%	69.80%
Y14d-R1	46,055,388	46,047,762	85.52%	80.13%	68.17%
Y14d-R2	55,236,996	55,216,088	83.15%	79.69%	64.92%
Y14d-R3	52,617,650	52,598,848	82.15%	80.72%	64.09%
B21d-R1	51,584,118	51,570,738	85.88%	79.34%	68.17%
B21d-R2	75,894,192	75,887,146	86.30%	80.81%	69.37%
B21d-R3	49,274,242	49,270,304	83.46%	82.07%	65.57%
Y21d-R1	48,639,404	48,633,740	85.79%	80.11%	68.07%
Y21d-R2	72,198,024	72,189,694	84.21%	80.53%	66.15%
Y21d-R3	62,312,324	62,301,528	82.57%	81.23%	64.55%
B28d-R1	51,826,640	51,812,730	82.41%	80.93%	64.73%
B28d-R2	62,519,596	62,507,548	81.30%	81.85%	63.27%
B28d-R3	54,378,064	54,368,498	83.34%	79.67%	66.58%
Y28d-R1	50,932,754	50,921,570	78.29%	81.74%	60.73%
Y28d-R2	68,581,712	68,572,206	78.90%	79.81%	60.73%
Y28d-R3	56,707,254	56,693,710	76.22%	80.77%	59.14%
B35d-R1	65,653,656	65,649,022	85.43%	75.92%	62.91%
B35d-R2	43,683,112	43,680,394	82.70%	81.01%	64.61%
B35d-R3	51,582,474	51,579,070	81.90%	81.29%	63.45%
Y35d-R1	61,880,872	61,875,512	83.17%	79.32%	64.26%
Y35d-R2	100,316,688	100,310,254	83.50%	80.52%	65.25%
Y35d-R3	52,007,020	52,003,094	82.36%	80.80%	64.26%
B42d-R1	47,870,940	47,844,452	77.41%	77.93%	59.53%
B42d-R2	66,974,066	66,964,418	81.39%	77.01%	61.06%
B42d-R3	65,652,928	65,645,534	80.58%	78.10%	60.27%
Y42d-R1	53,601,138	53,595,118	83.04%	77.31%	62.88%
Y42d-R2	61,128,968	61,122,232	84.13%	73.21%	60.59%
Y42d-R3	75,683,940	75,675,696	81.20%	78.39%	60.74%
Total	1,771,111,744	1,770,818,886			
Average	59,037,058	59,027,296	82.77%	79.60%	64.31%

[a] B, B-NIL; Y, Y-NIL; R1, R2 and R3, three biological replicates.
[b] Percent of reads in clean reads.
[c] Percent of reads in mapped reads.
